# *In vivo* miRNA knockout screening identifies miR-190b as a novel tumor suppressor

**DOI:** 10.1371/journal.pgen.1009168

**Published:** 2020-11-02

**Authors:** Hui Hong, Shun Yao, Yuanyuan Zhang, Yi Ye, Cheng Li, Liang Hu, Yihua Sun, Hsin-Yi Huang, Hongbin Ji

**Affiliations:** 1 Department of Thoracic Surgery, Fudan University Shanghai Cancer Center, Shanghai, China; 2 Department of Oncology, Shanghai Medical College, Fudan University, Shanghai, China; 3 State Key Laboratory of Cell Biology, Shanghai Institute of Biochemistry and Cell Biology, Center for Excellence in Molecular Cell Science, Chinese Academy of Sciences; Shanghai, China; 4 University of Chinese Academy of Sciences, Beijing, China; 5 BIOPIC and School of Life Sciences, Peking University, Beijing, China; 6 School of Life Science and Technology, Shanghai Tech University, Shanghai, China; 7 Peking-Tsinghua Center for Life Sciences, Academy for Advanced Interdisciplinary Studies, School of Life Sciences, Peking University, Beijing, China; 8 Center for Statistical Science, Center for Bioinformatics, Peking University, Beijing, China; Brigham and Women's Hospital, UNITED STATES

## Abstract

MicroRNAs (miRNAs) play important roles in the development of various cancers including lung cancer which is one of the devastating diseases worldwide. How miRNAs function in *de novo* lung tumorigenesis remains largely unknown. We here developed a CRISPR/Cas9-mediated dual guide RNA (dgRNA) system to knockout miRNAs in genetically engineered mouse model (GEMM). Through bioinformatic analyses of human lung cancer miRNA database, we identified 16 downregulated miRNAs associated with malignant progression and performed individual knockout with dgRNA system in *Kras*^*G12D*^*/Trp53*^*L/L*^ (*KP*) mouse model. Using this *in vivo* knockout screening, we identified miR-30b and miR-146a, which has been previously reported as tumor suppressors and miR-190b, a new tumor-suppressive miRNA in lung cancer development. Over-expression of miR-190b in *KP* model as well as human lung cancer cell lines significantly suppressed malignant progression. We further found that miR-190b targeted the *Hus1* gene and knockout of *Hus1* in *KP* model dramatically suppressed lung tumorigenesis. Collectively, our study developed an *in vivo* miRNA knockout platform for functionally screening in GEMM and identified miR-190b as a new tumor suppressor in lung cancer.

## Author summary

CRISPR/Cas9 has been widely used for genomic editing, especially for in vivo somatic gene mutation/fusion engineering in cancer research. Such technique provides the base for large scale screening of tumor suppressors in genetically engineered mouse model (GEMM). Up to date, no effective miRNA somatic knockout system has been reported yet, mainly ascribed to the imperfect matching mechanism for miRNA functional execution. Here we develop the dual guide RNA (dgRNA)-mediated CRISPR/Cas9 knockout system for the effective miRNA knockout in *Kras*^*G12D*^*Trp53*^*L/L*^ (*KP*) lung cancer mouse model. Through screening of 16 miRNAs down-regulated in human lung cancer, we identify multiple tumor-suppressive miRNAs: two previously reported miRNAs including miR-30b and miR-146a, and one novel miRNA miR-190b. We further demonstrate that miR-190b functions through targeting the *Hus1* gene to inhibit lung tumorigenesis. Taken together, our study develops the first in vivo miRNA knockout platform for functionally screening of tumor-suppressive miRNAs in GEMM and identifies the miR-190b as a new tumor suppressor in lung tumorigenesis.

## Introduction

Lung cancer is one of the most devastating diseases worldwide, accounting for about 24% of cancer-related death annually [1]. Although great progress has been achieved in lung cancer therapy, the overall 5-year survival rate still remains as low as approximately 19% [1]. Understanding the molecular mechanisms underlying lung tumorigenesis is important for the development of effective cancer treatment.

MicroRNAs (miRNAs) are a class of 18–23 nucleotide noncoding RNAs [2]. Dysregulation of miRNAs has been implicated in various types of cancers, such as lung cancer, prostate cancer, breast cancer as well as gastrointestinal cancer [2, 3]. MiRNAs participate in almost all the cellular activities, including apoptosis, cell proliferation, invasion, metastasis, and angiogenesis [4]. For example, miR-30b functions as a tumor suppressor through the inhibition of cell proliferation, metastasis and epithelial-to-mesenchymal transition in different carcinomas [5–8]. The miR-146a is down-regulated in lung cancer and frequently associates with distant organ metastasis and poor prognosis [9, 10]. Over-expression of miR-146 inhibits cell proliferation through targeting epidermal growth factor receptor (EGFR) [9, 11]. Additionally, many other tumor suppressive miRNAs affect tumor associated signaling pathways and drug resistance [4]. It’s worth noting that most of these studies have been done *in vitro* and/or in cell lines. Systematic identification of those important miRNAs using *in vivo* screening system remains an obstacle to gain deep understanding of the miRNAs’ function in lung tumorigenesis.

The CRISPR (clustered regularly interspaced short palindromic repeats)-Cas9 (CRISPR-associated 9) system is a powerful genome-editing tool [12], in which the Cas9 nuclease is directed by a guide RNA (gRNA) to the specific genome locus, creating a double-strand break (DSB), and then DNA is repaired by nonhomologous end-joining (NHEJ) [13, 14]. NHEJ repair results in insertions or deletions (in-dels) in the targeted locus and causes the loss of function if the DSB occurs in a coding region [13, 14]. The CRISPR-Cas9 system is highly efficient for systematic screening for oncogene and tumor suppressor genes (TSGs) *in vivo* [15–17]. For the miRNA functional study, miRNA inhibitors, which pair with and block native microRNAs through sequence complementarity are widely used [18]. This approach mainly provides a transient inhibition of microRNAs. Antagomirs are chemically modified, cholesterol-conjugated antisense oligonucleotides and have been demonstrated to silence certain miRNAs *in vivo* [19]. MiRNA sponges are also used to silence microRNAs in cell culture and transgenic animals [20]. For non-coding RNA knockout, CRISPR-Cas9 with two gRNAs has been applied to generate a relative larger genomic deletion *in vitro* [21–23]. Application of such technique *in vivo*, especially in genetically engineered mouse model (GEMM), will be very helpful to understand the function of miRNAs in lung tumorigenesis.

We here developed a CRISPR/Cas9-mediated dual guide RNA (dgRNA) approach for *in vivo* miRNA knockout and screened a serial of potential tumor-suppressive miRNAs individually in *Kras*^*G12D*^*/Trp53*^*L/L*^ (*KP*) mouse model. We identified three tumor suppressors including miR30b, miR146a and miR-190b, and further showed that miR-190b inhibited lung tumorigenesis potentially through targeting the *HUS1* gene.

## Results

### *In vivo* miRNA knockout screening identifies tumor suppressors in lung tumorigenesis

**Fig 1:**
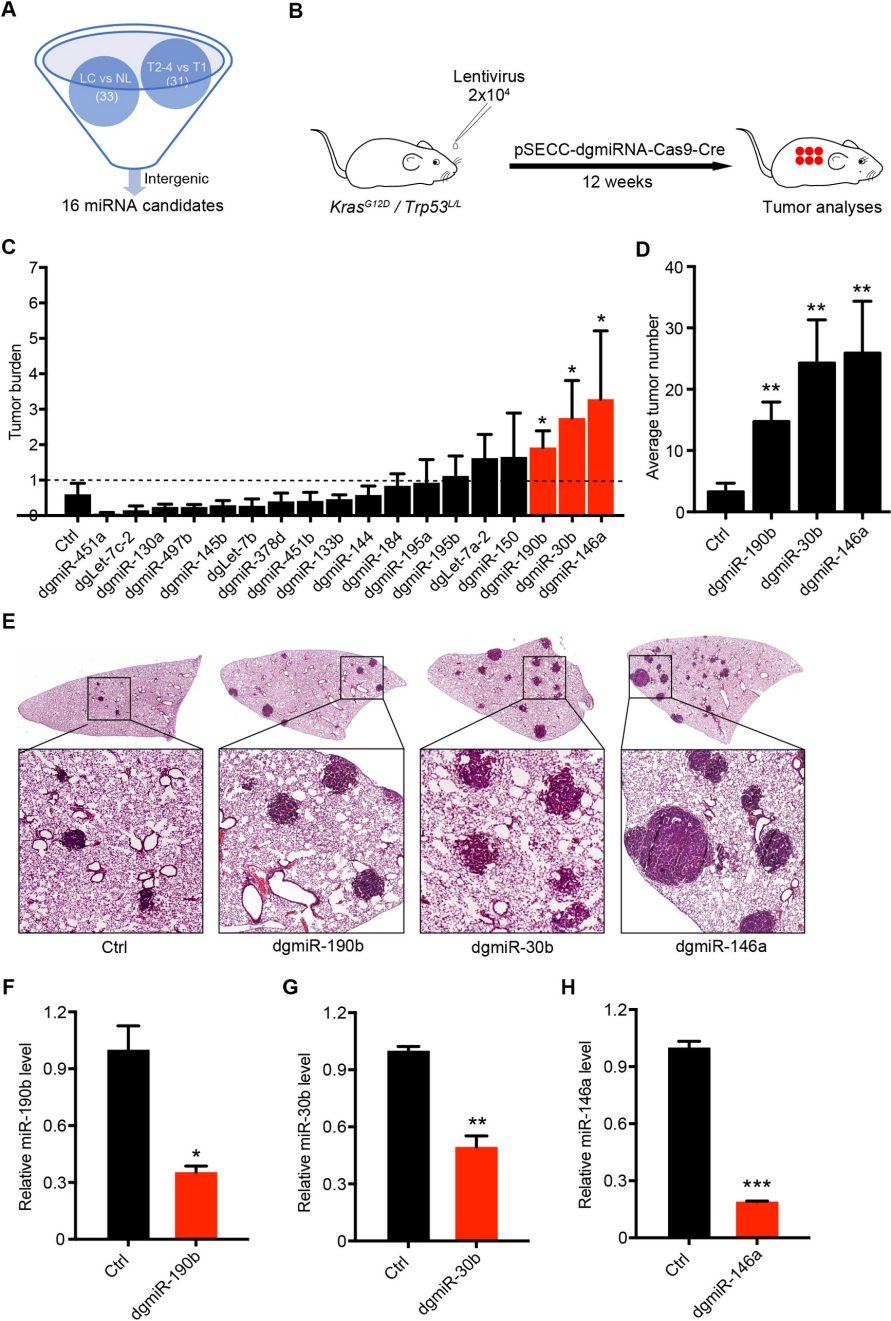
*In vivo* miRNA knockout screening identifies tumor suppressors in lung tumorigenesis. (A) Schematic illustration of potential miRNA identification through bioinformatics analysis. 33 means the number of miRNAs down-regulated in lung cancer (LC) compared with pathologically normal lung tissues (NL). 31 means the number of miRNAs down-regulated at late stages (T2-4) compared with early stage of T categories (T1). miRNAs located at the intergenic regions were further chosen for functional screening. (B) Schematic illustration of dual guide RNA (dgRNA) CRISPR/Cas9-meidiated knockout screening in *Kras*^*G12D*^*/Trp53*^*L/L*^ (*KP*) lung cancer mouse model. *KP* mice at 6–8 week-old were infected with 2×10^4^ PFU of pSECC-dgmiRNA lentivirus individually targeting 16 potential miRNA precursors (n = 3–15 mice for each group) and were analyzed 12 weeks later. (C) Average tumor burden of miRNA knockout in *KP* model. Data represent the mean ± SEM. Student’s *t* test; *P < 0.05. (D) Quantification of tumor numbers. Data represent the mean ± SEM. Student’s *t* test; **P < 0.01. (E) Representative photographs of hematoxylin and eosin (H&E) staining of mouse lungs from indicated groups. (F-H). Real-time quantitative PCR detection of miR-30b (F), miR-146a (G) and miR-190b (H) in the control and miRNA-knockout lung tumors. U6 served as an internal control. Data represent the mean ± SEM. Student’s *t* test; *P < 0.05; **P < 0.01; ***P < 0.001.

To identify tumor-suppressive miRNAs that are down-regulated in lung tumorigenesis, we first generated a list of miRNA candidates based on bioinformatic analyses of miRNA expression and TNM classification from the TCGA database (http://cancergenome.nih.gov/). According to the miRNA expression profile, we identified 33 miRNAs that were at least 2-fold down-regulated in lung cancer tissues compared with their adjacent normal tissues. We further identified 31 miRNAs which were down-regulated in late T categories stages (T2-4) compared with early stage (T1). We then excluded those miRNAs located at the gene coding regions to avoid the difficulty in functional interpretation during the miRNA knockout screening. This eventually gave us a list of 16 miRNAs located at the intergenic regions and their knockout wouldn’t affect neighborhood gene expression (Fig 1A and S1 Table).

As we know, miRNAs usually target genes through imperfect matching. Previous study has shown that dual guide RNA (dgRNA) instead of single guide RNA-mediated knockout might be optimal for miRNA knockout [24]. We therefore developed the CRISPR/Cas9-mediated dgRNA *in vivo* knockout system, in which miRNA precursor was targeted by two guide RNAs. Using this system, we individually knocked out the 16 miRNA precursors in the *KP* lung cancer mouse model. As previously described [17], the *KP* mice at 6–8 week-old were treated with 2×10^4^ PFU of lentivirus targeting indicated miRNA precursors individually through nasal inhalation (n = 5~8 mice for each miRNA knockout) (Fig 1B). 12 weeks afterwards, we analyzed the lung tumor formation. Pathological analyses showed that knockout of either miR-30b, miR-146a or miR-190b significantly promoted lung tumorigenesis, indicated by significantly increased tumor burden (Fig 1C). Consistently, tumor numbers were also significantly increased (Fig 1D and 1E). We further confirmed the decreased expression of 3 miRNAs through real-time PCR (Fig 1F–1H). Taken together, these data established the *in vivo* miRNA knockout system using dgRNA and identified potential tumor suppressors in lung tumorigenesis.

### Ectopic miR-190b expression inhibits lung tumorigenesis

**Fig 2:**
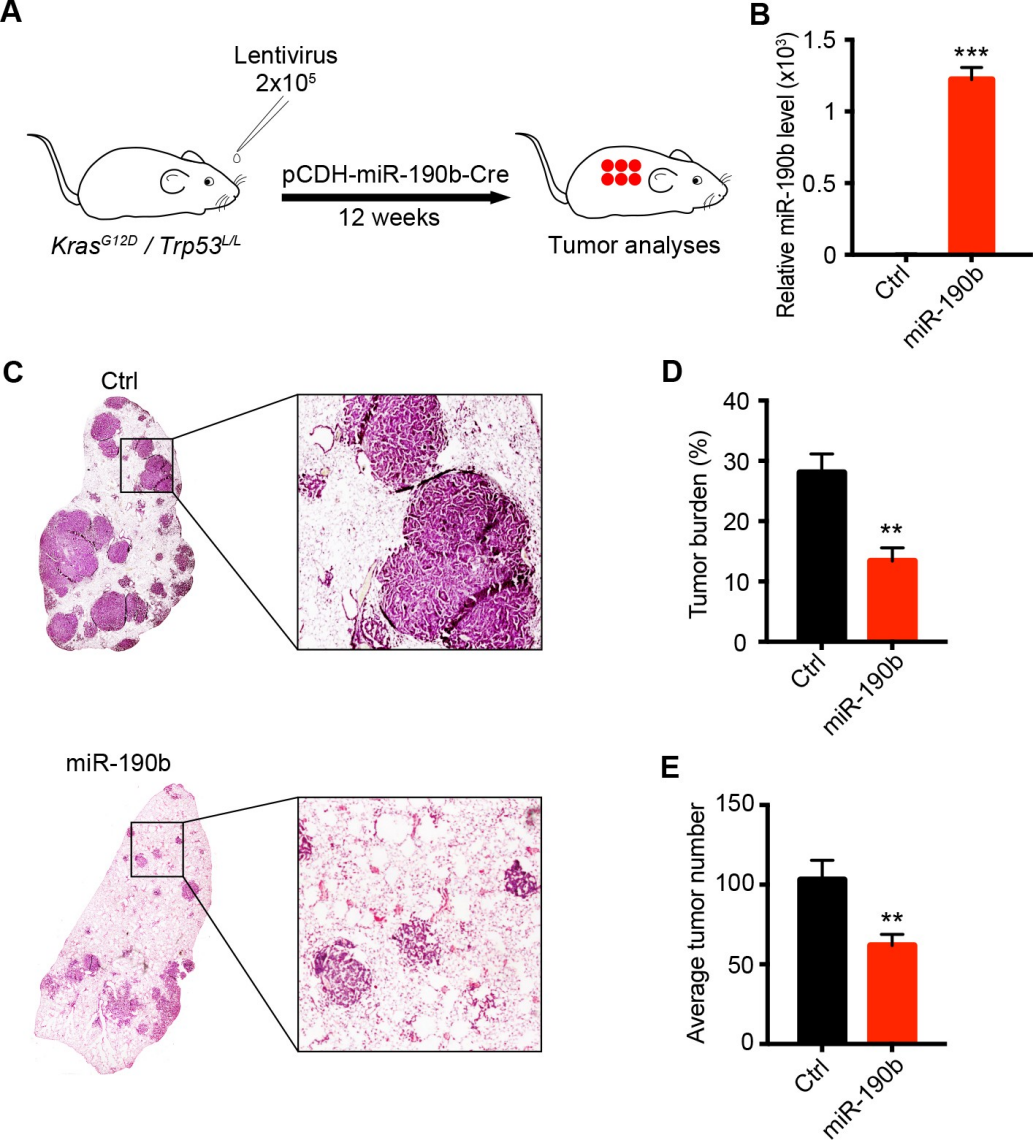
Ectopic miR-190b expression inhibits lung tumorigenesis. (A) Schematic illustration of miR-190b over-expression in *KP* lung cancer mouse model. *KP* mice at 6–8 weeks-old were infected with 2×10^5^ PFU of pCDH-miR-190b lentivirus (n = 6 mice) and were analyzed after 12 weeks. (B) Real-time quantitative PCR detection of miR-190b in the control and miR-190b over-expression lung tumors. U6 served as an internal control. Data represent the mean ± SEM. Student’s *t* test; ***P < 0.001. (C) Representative photographs of H&E staining of mouse lungs from indicated groups. (D)(E) Quantification of tumor burden (D) and tumor number (E). Data represent the mean ± SEM. Student’s *t* test; **P < 0.01.

Among three miRNA candidates, miR-30b and miR-146a have been well-documented as tumor suppressors in various cancers [5, 7, 9, 11, 25, 26]. This indirectly validated our in vivo miRNA knockout system. Interestingly, no previous functional study of miR-190b in lung tumorigenesis has been reported yet. We then chose miR-190b for further study. To functionally validate the tumor-suppressive function of miR-190b, we performed the over-expression of miR-190b precursor in the *KP* mouse model through nasal inhalation as previously described [27, 28]. After 12 weeks of nasal inhalation with 2×10^6^ PFU lentivirus, the *KP* mice were sacrificed for molecular and pathological analyses (Fig 2A). We first confirmed the ectopic miR-190b expression in *KP* lung tumors (Fig 2B). Pathological analyses clearly showed that miR-190b over-expression dramatically inhibited *KP* lung tumor formation (Fig 2C). Statistical analyses uncovered the obvious decrease of not only tumor burden but also tumor number (Fig 2C–2E). Together with dgRNA-mediated knockout study, these data strongly supported the tumor suppressive role of miR-190b in lung tumorigenesis.

### MiR-190b over-expression inhibits human lung cancer cell proliferation

**Fig 3:**
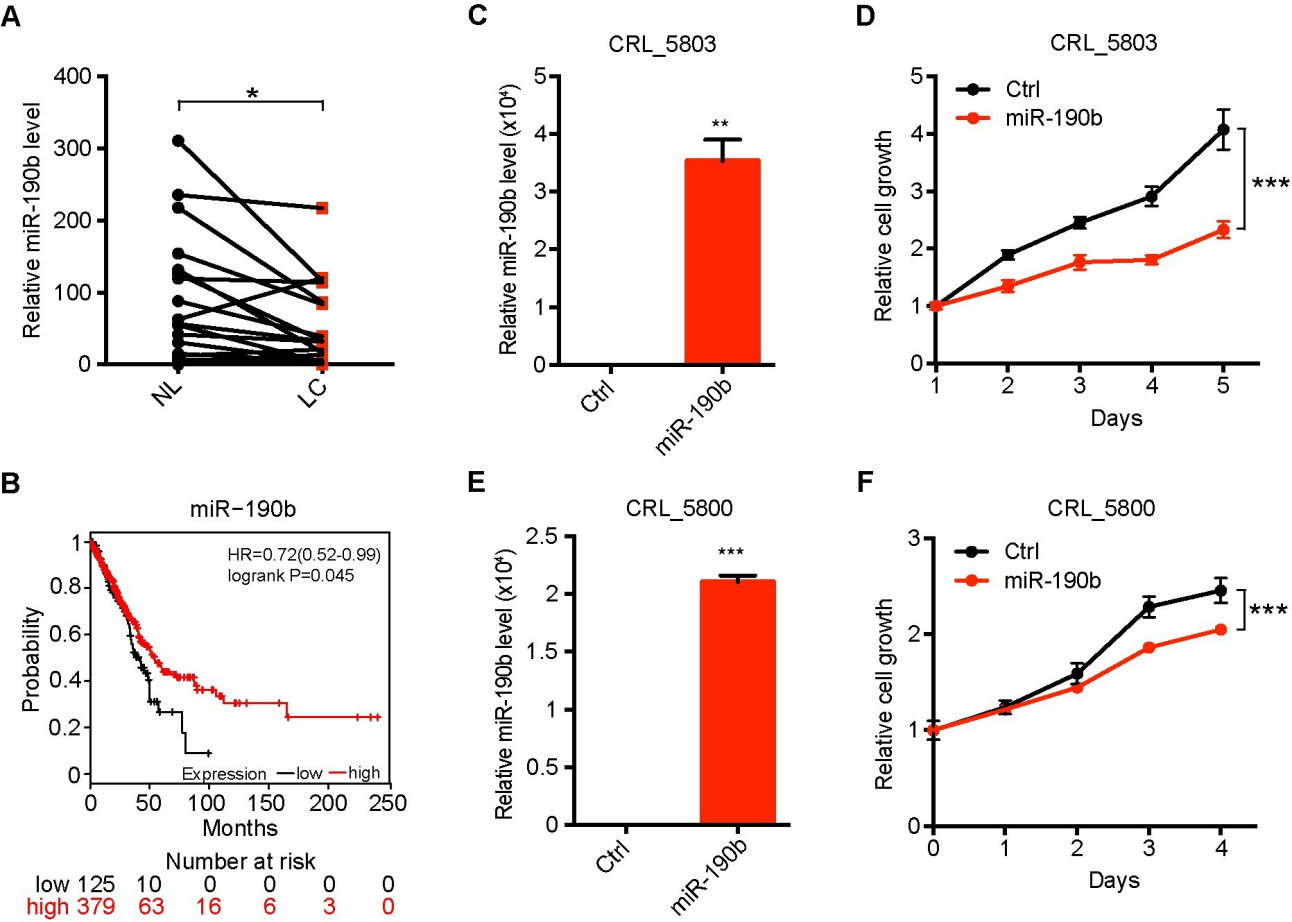
MiR-190b over-expression inhibits human lung cancer cell proliferation. (A) Real-time quantitative PCR detection of miR-190b in 25 human lung cancer specimens with adjacent pathologically normal lung tissues. U6 served as an internal control. Data represent the mean ± SEM. Student’s *t* test; *P < 0.05. (B) Kaplan-Meier plot analysis of miR-190b in lung cancer (http://kmplot.com/analysis/index.php?p=service&cancer=pancancer_mirna). (C)(D) The miR-190b was ectopically expressed in human lung cancer cell line CRL_5803. Real-time quantitative PCR detection of miR-190b was performed to detect the expression efficiency (C). The MTT assay was performed to detect cell proliferation rate (D). Data represent the mean ± SEM. Student’s *t* test; ***P < 0.001. (E)(F) The miR-190b was ectopically expressed in human lung cancer cell line CRL_5800. Real-time quantitative PCR of miR-190b was performed to detect the over expression efficiency (E). The MTT assay was performed to detect cell proliferation rate (F). Data represent the mean ± SEM. Student’s *t* test; ***P < 0.001.

Next, we assessed the expression levels of miR-190b in Chinese lung cancer samples in comparison with adjacent pathologically normal lung tissues. Consistent with TCGA data, our real-time PCR data showed that miR-190b expression was frequently down-regulated in Chinese lung cancer samples (Fig 3A). Due to limited sample number analyzed for miR-190b expression, we further took advantage of Kaplan-Meier Plotter (http://kmplot.com/analysis/index.php?p=service&cancer=pancancer_mirna) for patient survival analyses [29]. The data analyses showed that low miR-190b expression was significantly associated with poor prognosis (Fig 3B).

We further evaluated the function of miR-190b using 2 human non-small cell lung cancer (NSCLC) cell lines CRL-5803 and CRL-5800. When miR-190b was over-expressed (Fig 3C–3E), both CRL-5803 and CRL-5800 cells displayed significantly decreased proliferation (Fig 3D–3F). These data from human lung cancer specimens and cell lines were consistent with the findings from GEMM, which further supported the tumor-suppressive role of miR-190b.

### *HUS1* is the downstream target of miR-190b

**Fig 4:**
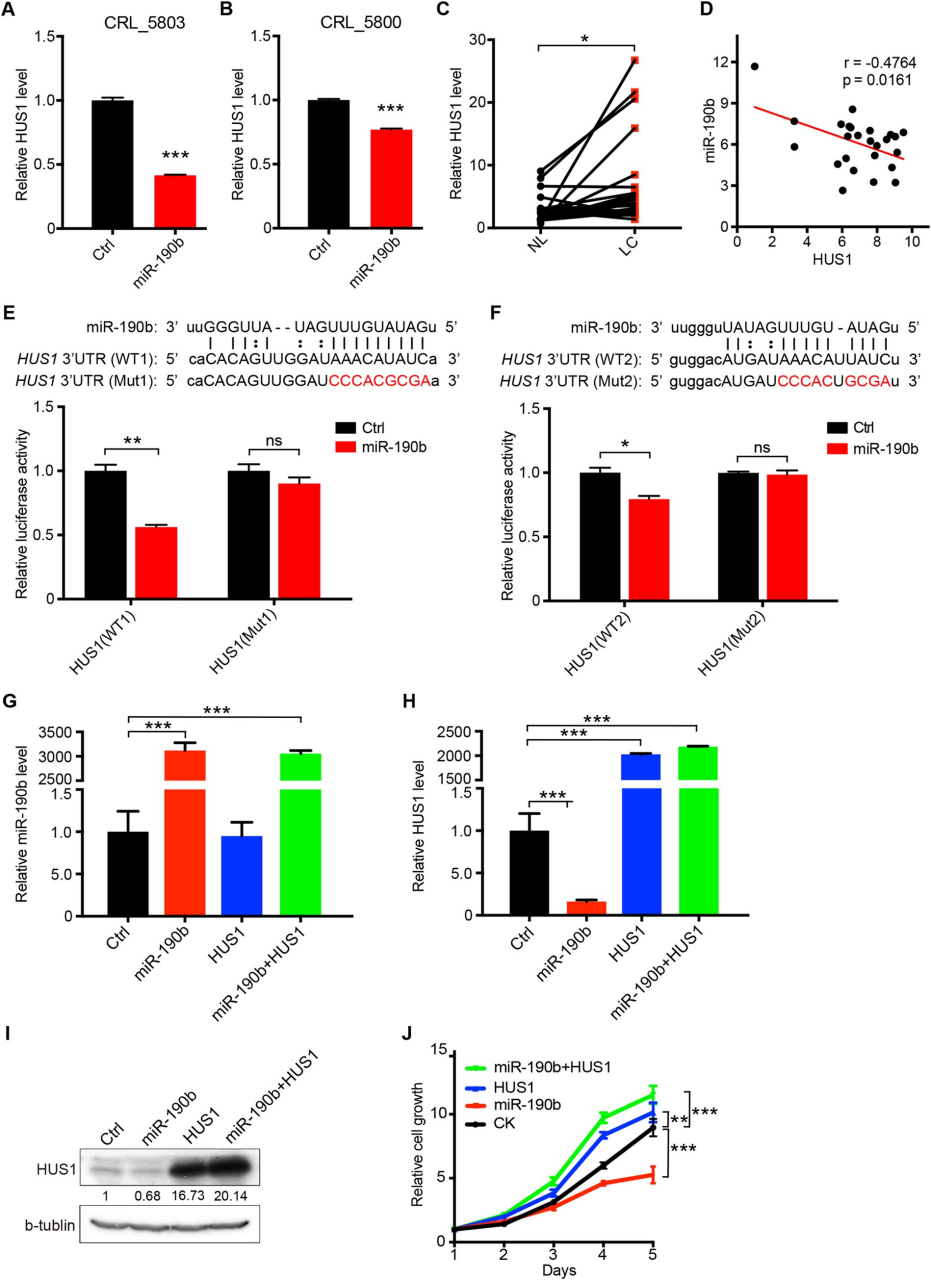
HUS1 is the downstream target of miR-190b. (A)(B) The expression level of HUS1 in miR-190b-overexpression cell lines CRL_5803 (A) and CRL_5800 (B) were assayed by real-time quantitative PCR. Data represent the mean ± SEM. Student’s *t* test; ***P < 0.001. (C) Real-time quantitative PCR of HUS1 in 25 human lung cancer samples compared with adjacent pathologically normal lung tissues. β-actin served as the internal control. Data represent the mean ± SEM. Student’s *t* test; *P < 0.05. (D) Correlation analysis of miR-190b and HUS1 expression level by linear regression (n = 25). (E)(F) Two predicted miR-190b binding sites in the 3′UTR of the HUS1 gene were shown with the corresponding sequence in the mutated version (Mut1 and Mut2). Dual luciferase assay in HEK-293T cells expressing miR-190b were transfected with the reporter constructs. Data represent the mean ± SEM. Student’s *t* test; *P < 0.05; **P < 0.01, ns: not significant. (H-J) CRL_5803 cells expressing miR-190b were further transfected with HUS1 over-expression vectors. The expression level of miR-190b (H) and HUS1 (I) were assayed by qPCR in indicated cell lines. The MTT assay was performed to detect cell proliferation rate (J). Data represent the mean ± SEM. Student’s *t* test; ***P < 0.001; ns: not significant.

MiRNAs inhibit gene expression mainly through interfering the stability and/or the translation of messenger RNAs (mRNAs) [30]. Using TargetScan database (http://www.targetscan.org/), we found that *HUS1*, a member of the RAD9-RAD1-HUS1 complex, is a potential target of miR-190b. Aberrant expression of this complex component frequently leads to tumorigenesis of the lung, breast, skin, thyroid and prostate [31–33]. Additionally, HUS1 is highly expressed in lung cancer and also gains gene copy numbers (S1A and S1B Fig). So we further focused on the function of miR-190b-Hus1 axis. To determine the regulation of HUS1 by miR-190b, we ectopically expressed miR-190b in CRL-5803 and CRL-5800 cells. We found that ectopic miR-190b expression clearly down-regulated the *HUS1* mRNA expression (Fig 4A and 4B). This indicated the potential negative regulation of HUS1 by miR-190b. Interestingly, *HUS1* expression was frequently up-regulated in Chinese lung cancer samples compared to adjacent pathologically normal lung tissues (Fig 4C). The potential regulation of HUS1 by miR-190b was further supported by the negative correlation in Chinese clinical cancer samples, but not in the TCGA database (Fig 4D).

Through bioinformatics analyses, we found 2 and 3 putative miR-190b-binding sites in the 3’UTR of human and mouse *HUS1* gene. To further determine whether HUS1 is the direct target of miR-190b, we constructed luciferase reporters containing either wild-type (WT) or mutant (Mut) miR-190b-binding sites in the 3’UTR of human and mouse *HUS1* genes, respectively. We found that ectopic miR-190b expression resulted in a significant reduction of luciferase activity in the WT reporters. However, this reduction was blocked when these binding sites were mutated (Fig 4E–4F, and S2 Fig). Consistent with this, the Hus1 expression was specifically up-regulated in miR-190b knockout KP tumors and down-regulated in miR-190b over-expression KP tumors (S3 and S4 Figs). Importantly, we found that ectopic HUS1 expression could rescue the inhibitory effect of miR-190b on cell proliferation (Fig 4G–4J). These data together demonstrated that miR-190b inhibited lung cancer cell proliferation potentially through targeting HUS1.

### Depletion of HUS1 inhibits *de novo* lung tumorigenesis

**Fig 5:**
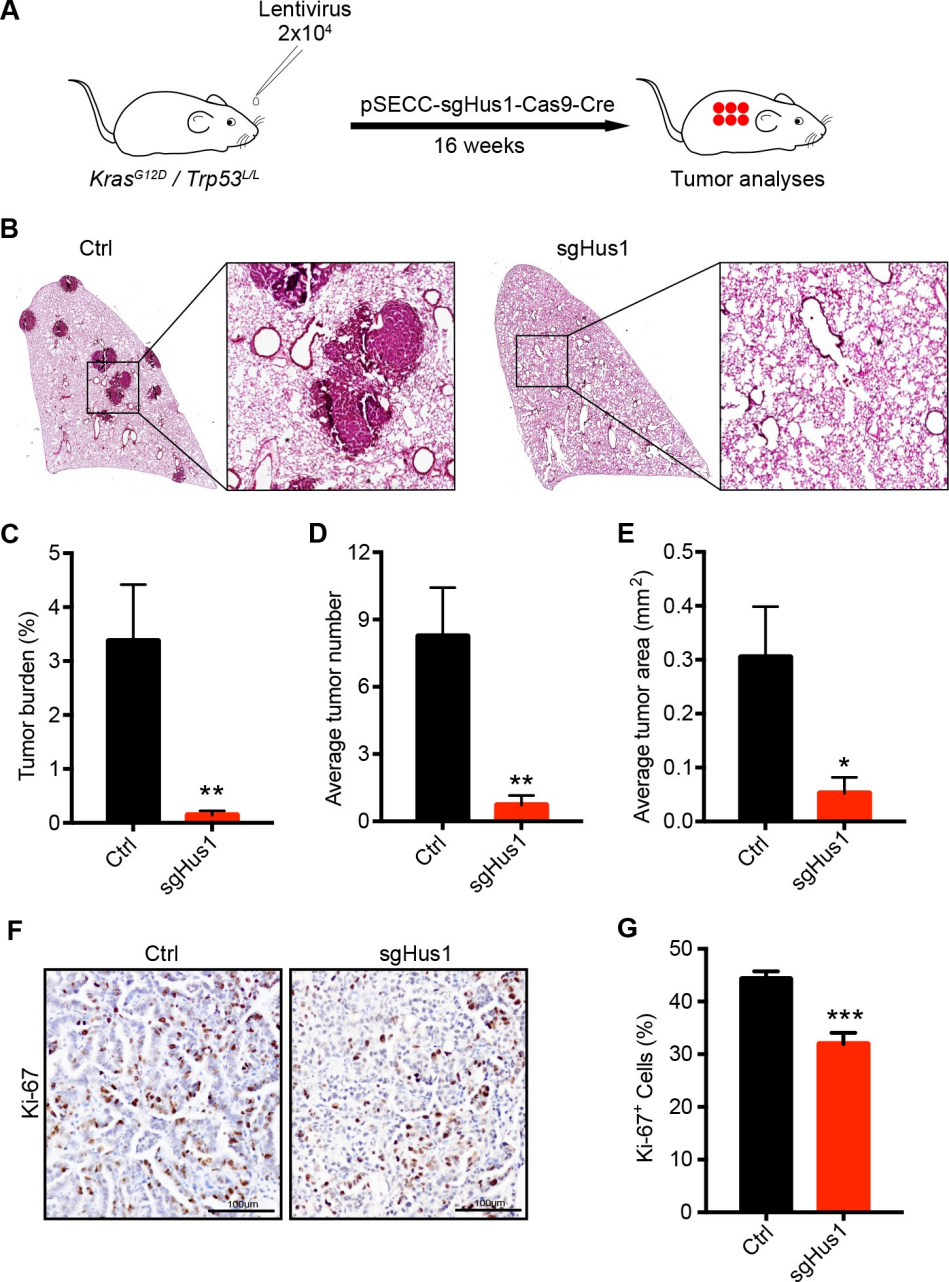
Depletion of HUS1 inhibits *de novo* lung tumorigenesis. (A) Schematic illustration of *HUS1* gene knockout in *KP* lung cancer mouse model. *KP* mice at 6–8 weeks-old (sgTomato, n = 13; sg*Hus1*, n = 10) were infected with 2×10^4^ PFU of pSECC-sg*Hus1* lentivirus and were analyzed after 16 weeks. (B) Representative photographs of H&E staining of mouse lungs from indicated groups. (C-E) Quantification of tumor burden (C), tumor number (D) and average tumor area (E). Data represent the mean ± SEM (n = 13 for sgTOM, n = 10 for sg*Hus1*). Student’s *t* test; *P < 0.05; **P < 0.01. (F) Representative photos of Ki-67 immunostaining in HUS1 knockout mice lung tumors. (G) Statistic of Ki-67 signal density of HUS1 knockout mice lung tumors. Student’s *t* test; ***P < 0.001.

Our data have indicated the promotive role of HUS1 in lung tumorigenesis. To further test this, we performed HUS1 knockout using traditional sgRNA-mediated knockout system in *KP* model as previously described [17]. After 12 weeks of nasal inhalation of 2×10^4^ PFU lentivirus targeting HUS1, the *KP* mice were analyzed (Fig 5A) and the knockout efficacy of Hus1 was assessed by IHC and DNA sequencing (S5 Fig). Pathological analyses uncovered a drastic inhibition of tumor formation by HUS1 knockout in *KP* model (Fig 5B). Almost no lung cancer was visible when HUS1 was knocked out (Fig 5B). Statistical analyses of the tumor burden, number and area further supported the essential role of HUS1 in lung tumorigenesis (Fig 5C–5E). Consistently, a significant decrease of tumor growth was found in the sg*Hus1* group indexed by Ki-67 immunostaining (Fig 5F and 5G).

## Discussion

Systematic screening for tumor-suppressive miRNAs critically involved in lung cancer development is of great importance. CRISPR/Cas9 technology has been widely applied for screening genes *in vitro* and *in vivo* [15, 34]. Here, we established a CRISPR/Cas9-mediated miRNA knockout system using dual guide RNAs and performed the miRNA functional screening in lung cancer mouse model. Such screening has eventually identified three miRNAs (miR-30b, miR-146a and miR-190b) negatively regulating lung tumorigenesis. Previous studies have established the functional link between miR-146a and miR-190b and lung cancer, indicating that these two miRNAs might serve as diagnostic biomarkers [10, 35, 36]. The miR-190b is previously shown to be up-regulated in ERα-positive breast tumors compared to ERα-negative breast tumors [37]. Recently, a plasma microRNA panel containing miR-190b, miR-17, and miR-375 is reported to have a high diagnostic accuracy in the discrimination of non-small cell lung cancer (NSCLC) and small cell lung cancer (SCLC) [38, 39]. However, little is known about the function of miR-190b in lung tumorigenesis. Our study here shows that miR-190b is frequently down-regulated in human lung cancer. Importantly, our *in vivo* loss-of-function as well as gain-of-function experiments clearly suggests a tumor-suppressive role of miR-190b in lung tumorigenesis.

The HUS1 checkpoint clamp component (HUS1) is a member of the RAD9-RAD1-HUS1 complex, which is loaded onto DNA under replicative stress and serves as a scaffold for the downstream factors which lead to cell-cycle arrest [40, 41]. Moreover, the RAD9-RAD1-HUS1 complex functions in DNA damage repair and telomere maintenance [42–44]. Aberrant expression of this complex component frequently leads to tumorigenesis of the lung, breast, skin, thyroid and prostate [31–33]. Our *in vitro* data suggest that HUS1 is the direct target of miR-190b. We further show that knockout of HUS1 in *KP* model clearly inhibits lung cancer malignant progression. This provides *in vivo* evidence to demonstrate the essential role of HUS1 in lung tumorigenesis. Moreover, ectopic expression of HUS1 *in vitro* can effectively reverse the inhibitory effect of miR-190b. Of course, it’ll be great if such rescue experiment could be done *in vivo*. However, due to the difficulty in viral packaging and infection, we failed to perform double knockouts of the miR-190b and Hus1 in *KP* model, which requires three guide RNAs. Future efforts might be necessary to test this using *KP* model together with either miR-190b or Hus1 conditional knockout mice. Nonetheless, clinical analyses show that *HUS1* is inversely correlated with miR-190b expression in Chinese human lung cancer. This indirectly supports the potential regulation of HUS1 by miR-190b. HUS1 expression doesn’t show an inverse correlation with miR-190b in TCGA samples. We think the possible reasons for not seeing negative correlations are as follows: patients from different regions may be different in clinical composition and the heterogeneity between different patient samples may affect the results. Besides, the expression of HUS1 in tumors may also be regulated by other factors. Our work mainly confirms that HUS1 is a functional target of miR-190b, and we cannot rule out the possibility that HUS1 may also be regulated by other miRNAs.

Together these findings support an oncogenic property of HUS1 in lung tumorigenesis. Previous study shows that HUS1 is a potential tumor suppressor in primary hepatocellular carcinoma [33]. On the contrary, downregulation of HUS1 by antisense oligonucleotides enhances the sensitivity of human lung carcinoma cells to cisplatin [45] and high HUS1 expression is significantly correlated with poor prognostic clinicopathologic factors in ovarian cancer [32]. Thus, the potential role of HUS1 as a tumor suppressor or tumor promoter may be organ-specific and varies with the types of malignancy. Future efforts will also be necessary to clarify this in more details.

## Materials and methods

### Ethics statement

For studies using human data, the study was approved by ethics committee of Fudan University Shanghai Cancer Center Institutional Review Board (approval number: 090977–1). Informed consents of all patients for donating their samples to the tissue bank of Fudan University Shanghai Cancer Center were obtained from patients themselves or their relatives.

Mice were housed in a specific pathogen-free environment at the Shanghai Institute of Biochemistry and Cell Biology and treated in accordance with protocols conformed to the ARRIVE guidelines and approved by the Institutional Animal Care and Use Committee of the Shanghai Institutes for Biological Sciences, Chinese Academy of Sciences (approval number: IBCB0011).

### Bioinformatic analysis

The miRNA expression and clinical outcome datasets were downloaded from the TCGA (http://cancergenome.nih.gov/), which contained Tumor stage (T stage, T1-4) information. We used 279 samples for T1 group and 720 samples for T2-4 group. The two-tailed t-test and fold change were performed to identify the differentially expressed miRNAs. MiRNAs with an adjusted P-value <0.05 after Benjamini & Hochberg correction and absolute fold change ≥2 were considered as differentially expressed miRNAs. The wilcoxon test was performed to identify differentially expressed miRNAs in late T categories stages (T2-4) compared with early stage (T1). MiRNAs with an adjusted P-value <0.05 were considered as differentially expressed miRNAs. miRNAs located at the intergenic region were chosen for further study. For Kaplan-Meier plot analysis of miR-190b in lung cancer, we used the 513 lung adenocarcinoma patients from TCGA database in http://kmplot.com/analysis/index.php?p=service&cancer=pancancer_mirna [29].

### Human lung cancer samples

A total of 25 human lung tumor specimens collected from Fudan University Shanghai Cancer Center were used for miR-190b and HUS1 expression analyses.

### Plasmid construction

For gene expression in cell lines, the precursors of miR-190b and the CDS of *HUS1* were PCR amplified and cloned into pCDH-puro vector. For miR-190b over-expression in KP mouse, miR-190b precursor was cloned into pCDH-Cre vector. For HUS1 gene knockout, the sgRNA of HUS1 was cloned into BsmBI sites of pSECC vector which contains both Cre and Cas9. For dual gRNA CRISPR screening, we insert another U6-filler-TA into pSECC, thus two gRNAs can be cloned into one vector. Briefly, the first gRNA was cloned into BsmBI sites of pSECC vector. For the second gRNA, the U6-filler-TA sequence was amplified from pSECC and inserted into the vector pCDNA3.1 between EcoRI and XhoI which we then called pCDNA3.1-linker. The second gRNA was cloned into BsmBI sites of pCDNA3.1-linker and then the U6-gRNA2-TA was cloned into pSECC-gRNA1 using ECoRI and XhoI. For the luciferase assay, the wild type 3′UTR of *HUS1* was cloned into pMIR-Report which is a construct containing firefly luciferase as a reporter. *HUS1* 3′UTR mutant constructs were obtained using Phusion Site-Directed Mutagenesis Kit (ThermoFisher). All primers used are listed in S2 Table.

### Cell culture

CRL-5803 and CRL-5800 were purchased from American Type Culture Collection (ATCC) and cultured in RMPI1640 (10-040-CV, Corning) supplemented with 8% fetal bovine serum (FBS). HEK-293T cells were all cultured in Dulbecco’s modified Eagle’s medium (DMEM) (10-017-CV, Corning) supplemented with 8% FBS. All the mediums were supplemented with 100 units/ml penicillin and 100 μg/ml streptomycin.

### Cell proliferation

For 3-(4,5-dimethylthiazol-2-yl)-2,5-diphenyltetrazolium bromide (MTT) assays, 2000 cells were seeded on 96-well plates, and the viability of cells was measured daily for 5 days. All experiments were performed in triplicate.

### Lentivirus production and infection

Brief description of lentivirus production protocol was reported according to the previously described procedures [17]. Cells infected with lentivirus were then selected by appropriate concentration of puromycin.

### Mouse colony, treatment, and tumor analysis

For CRISPR/Cas9 screening, *Kras*^*G12D*^*/Trp53*^*L/L*^ mice at the age of 6–8 week-old were treated with 2×10^4^ PFU of pSECC-dgmiRNAs lentivirus via nasal inhalation (n = 3–15 mice for each group) as previously described [28] and analyzed after 12 weeks. For miR-190b over-expression, *KP* mice at the age of 6–8 week-old were treated with 2×10^5^ PFU of pCDH-miR-190b lentivirus via nasal inhalation (n = 6 for each group) and analyzed 12 weeks afterwards. For *Hus1* knockout, *KP* mice at the age of 6–8 week-old were treated with 2×10^4^ PFU of pSECC-sg*Hus1* lentivirus via nasal inhalation (sgTomato, n = 13; sg*Hus1*, n = 10). After 16 weeks, tumor numbers and burden were analyzed as previously described [28]. Briefly, the tumor numbers of five lobes were counted in each mouse. Tumor burden was analyzed as the follows: photos of HE staining sections were taken by microscope. Then tumor area and total lung area of five lobes in each mouse were determined through Image J software. Tumor burden is defined by the ratio of tumor area and total lung area.

### Quantitative real-time PCR

Total RNA was extracted using TRIzol reagent (Invitrogen) and retrotranscribed into first-strand cDNA using PrimeScript RT reagent Kit with gDNA Eraser (TaKaRa). The cDNAs were subjected to real-time PCR with gene-specific primers using SYBR-Green Master PCR mix (Roche). Actin was served as internal controls. For miRNA real-time PCR, an adaptor was added to the 5’ of the reserve primer as listed in S2 Table. The protocol of qRT-PCR for miRNA was conducted as previously described [46, 47]. U6 was used as the internal controls.

### Hematoxylin-Eosin staining (H&E)

H&E was performed as previously described [48]. Briefly, mice were sacrificed and lung tissues were inflated and fixed in 10% formalin, embedded in paraffin, sectioned at 5-μm thickness, and then stained with hematoxylin and eosin.

### Luciferase assay

HEK-293T cells were seeded in 24-well plates at a density of 1.5×10^5^ cells/well and cultured for 24 hrs. In the luciferase reporter gene assay, The pMIR-Report clones were co-transfected with pCDH-miR-190b and pRL-TK-Renilla (Promega E2241) into HEK-293T cells. After 48 hours, the luciferase activity was measured. Cells were washed with 1xPBS and lysed. Dual Luciferase Reporter Assay System (Promega, Madison, USA) was used to measure the luciferase and renilla activities. All the luciferase values were normalized to that of the Renilla values and the ratio of firefly/renilla was presented.

### Statistical analysis

Differences were compared using Student’s t-test. P value<0.05 was considered statistically significant. P values of each experiment were included in the figure legends. Error bars were represented with SEM, described in each figure legends. All analyses were performed with Graph Pad Prism 5 software.

## Supporting information

S1 Fig HUS1 expression, copy number variation and its correlation with miR-190b in human lung cancer. (A) HUS1 expression in LUAD from TCGA database. (B) Pie chart of HUS1 copy number variation in Chinese lung cancer patients [17]. CN: Copy Number. The number of patients with different copy number are shown in the pie chart. Numbers in the bracket shows the percentage of patients with different copy number. (C) Expression correlation of HUS1 and miR-190b from TCGA database.
(TIF)

S2 Fig Mouse Hus1 is targeted by miR-190b. (A) Three potential miR-190b binding sites in the 3′UTR of mouse Hus1 gene were shown with the corresponding mutants (Mut1, Mut2 and Mut3). (B) Dual luciferase assay in HEK-293T cells expressing miR-190b were transfected with the reporter constructs as indicated. Data were presented as mean ± S.E.M. Student’s *t* test; *P < 0.05; ***P < 0.001, ns: not significant.
(TIF)

S3 Fig Hus1 is up-regulated in miR-190b knockout KP mouse. (A) Real-time quantitative PCR detection of Hus1 in the control and miRNA-knockout lung tumors. Actin served as internal control. Data were presented as mean ± S.E.M. Student’s *t* test; **P < 0.01; ns: not significant. (B) Representative photographs of hematoxylin and eosin (H&E) staining of mouse lungs from indicated groups.
(TIF)

S4 Fig Hus1 is suppressed in miR-190b-overexpression KP mouse. (A) Real-time quantitative PCR detection of Hus1 in control and miR-190b over-expression lung tumors. Actin served as internal control. Data were presented as mean ± S.E.M. Student’s *t* test; *P < 0.05. (B) Representative photographs of hematoxylin and eosin (H&E) staining of mouse lungs from indicated groups.
(TIF)

S5 Fig Knockout efficacy of sgHus1. (A) Representative photographs of hematoxylin and eosin (H&E) staining of mouse lungs from control (sgTOM) and sgHus1 KP mouse. (B) The on-target efficiency of sgRNA-Hus1 in control and sgHus1 KP13 cell line.
(TIF)

S1 Table A list of 16 tumor-suppresive miRNA candidates in human lung cancer. (PDF)

S2 Table Primers used for this study. (PDF)
