# Supplementary figures and images for: In vivo miRNA knockout screening identifies miR-190b as a novel tumor suppressor
Source: PLoS Genet. 2020 Nov 2;16(11):e1009168. doi: 10.1371/journal.pgen.1009168 (PMC7660552; doi:10.1371/journal.pgen.1009168)

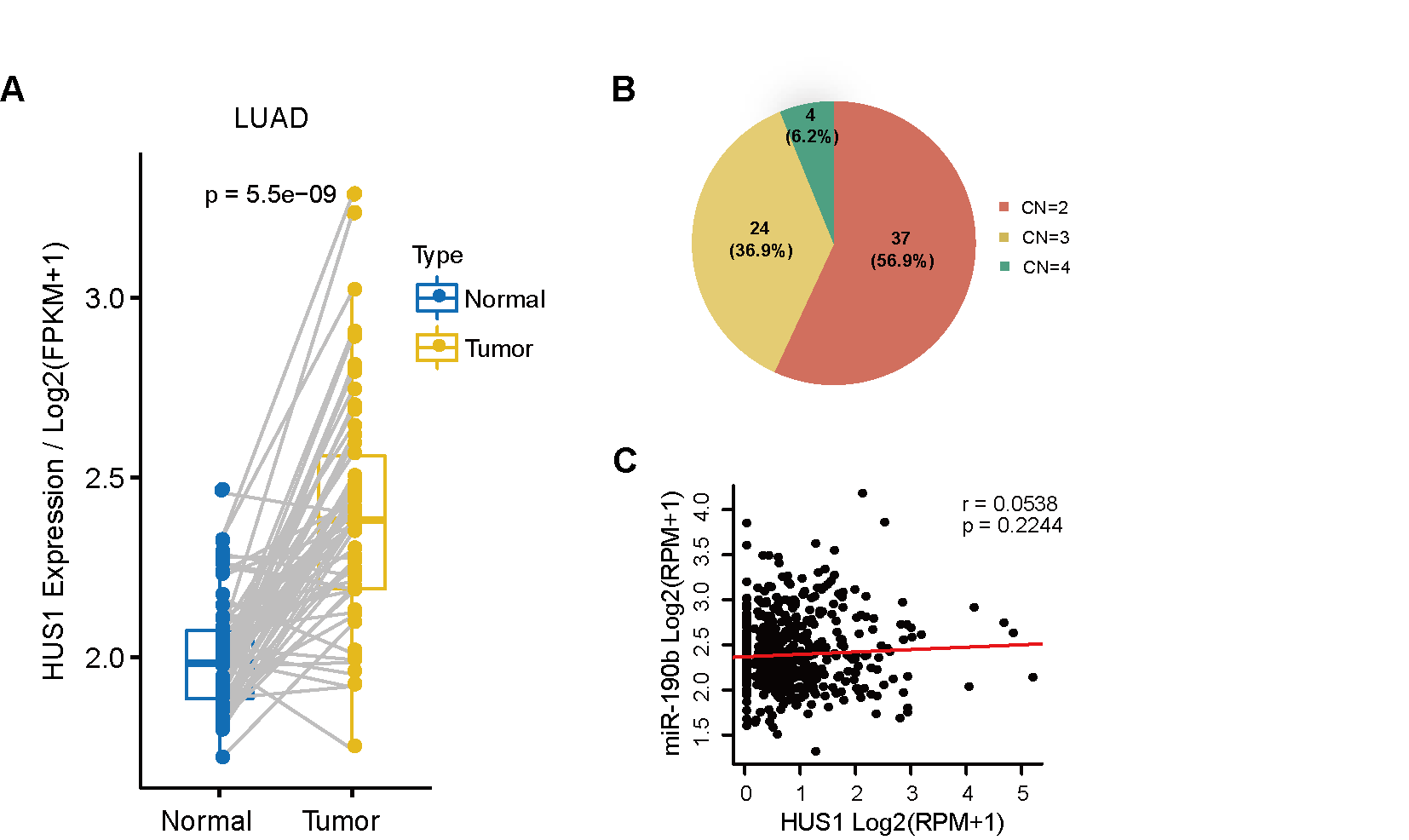

Supplement: S1 Fig — (A) HUS1 expression in LUAD from TCGA database. (B) Pie chart of HUS1 copy number variation in Chinese lung cancer patients [17]. CN: Copy Number. The number of patients with different copy number are shown in the pie chart. Numbers in the bracket shows the percentage of patients with different copy number. (C) Expression correlation of HUS1 and miR-190b from TCGA database. (TIF) [file pgen.1009168.s001.tif]

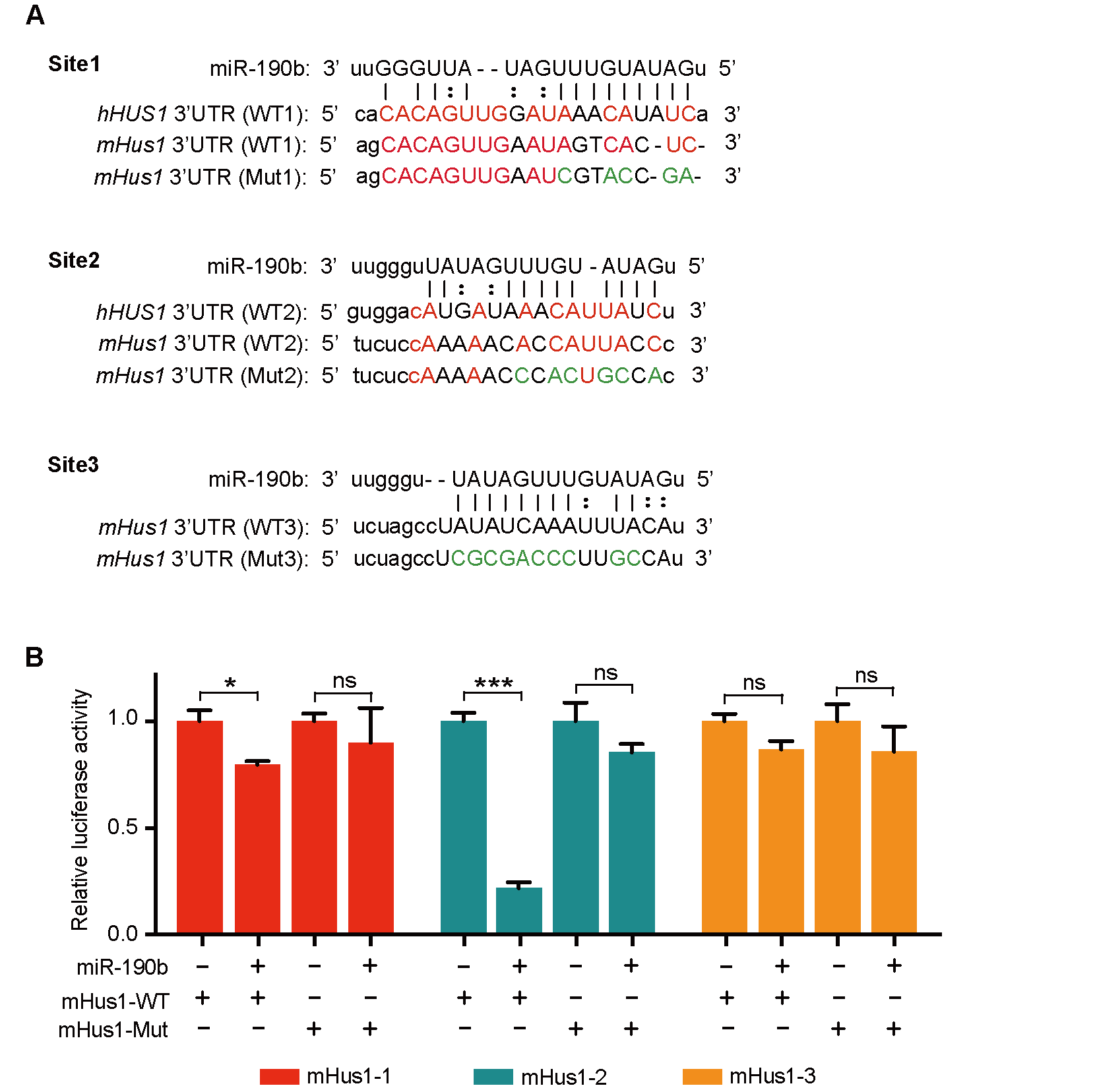

Supplement: S2 Fig — (A) Three potential miR-190b binding sites in the 3′UTR of mouse Hus1 gene were shown with the corresponding mutants (Mut1, Mut2 and Mut3). (B) Dual luciferase assay in HEK-293T cells expressing miR-190b were transfected with the reporter constructs as indicated. Data were presented as mean ± S.E.M. Student’s t test; *P < 0.05; ***P < 0.001, ns: not significant. (TIF) [file pgen.1009168.s002.tif]

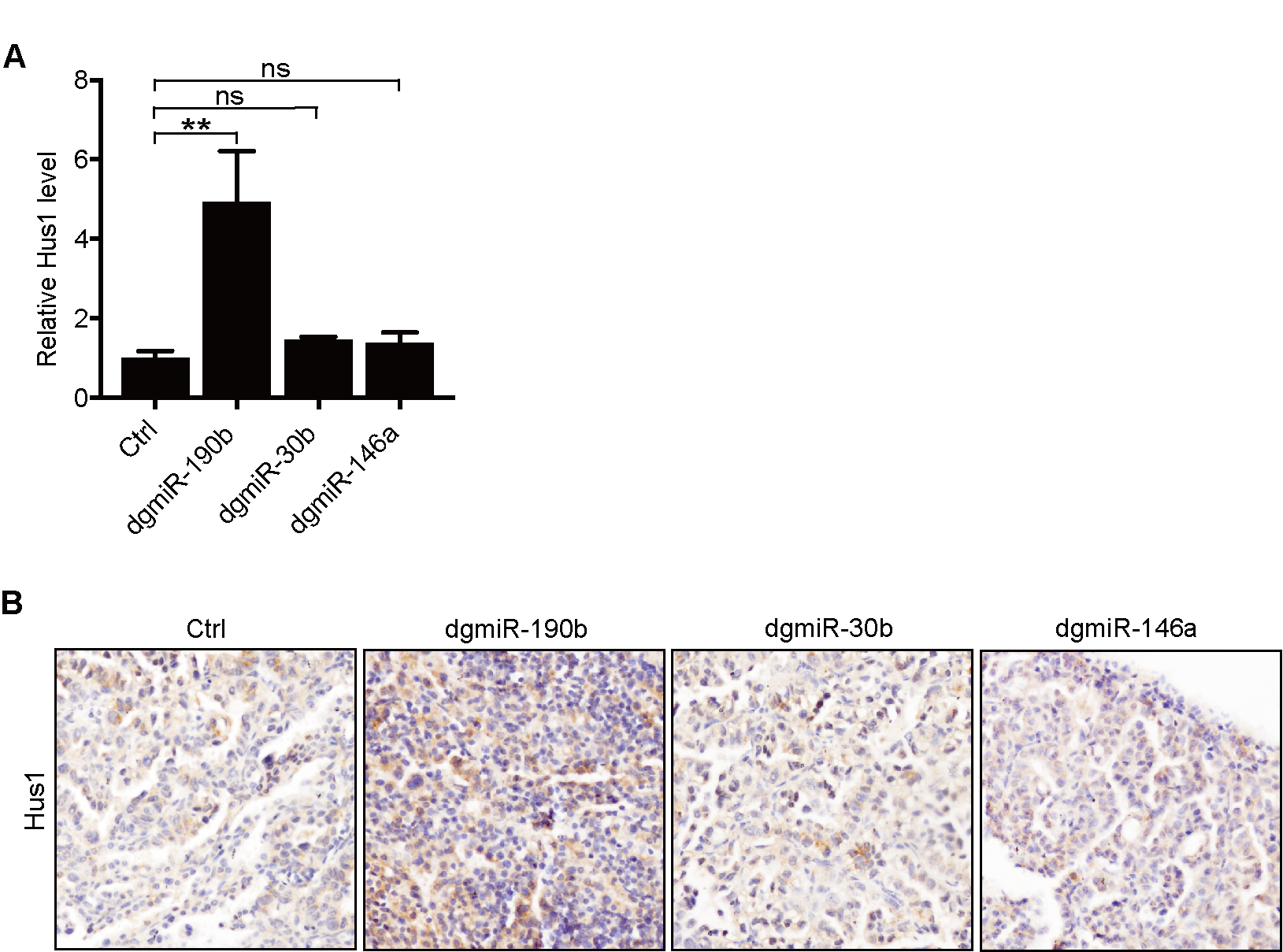

Supplement: S3 Fig — (A) Real-time quantitative PCR detection of Hus1 in the control and miRNA-knockout lung tumors. Actin served as internal control. Data were presented as mean ± S.E.M. Student’s t test; **P < 0.01; ns: not significant. (B) Representative photographs of hematoxylin and eosin (H&E) staining of mouse lungs from indicated groups. (TIF) [file pgen.1009168.s003.tif]

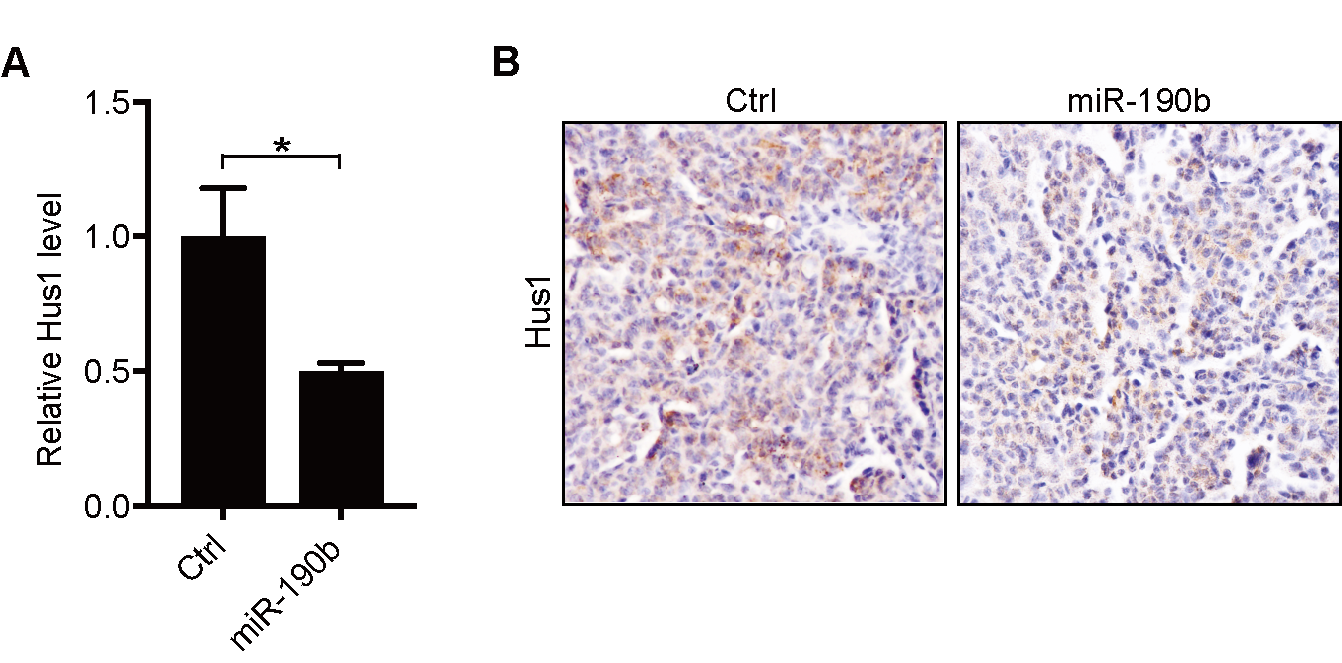

Supplement: S4 Fig — (A) Real-time quantitative PCR detection of Hus1 in control and miR-190b over-expression lung tumors. Actin served as internal control. Data were presented as mean ± S.E.M. Student’s t test; *P < 0.05. (B) Representative photographs of hematoxylin and eosin (H&E) staining of mouse lungs from indicated groups. (TIF) [file pgen.1009168.s004.tif]

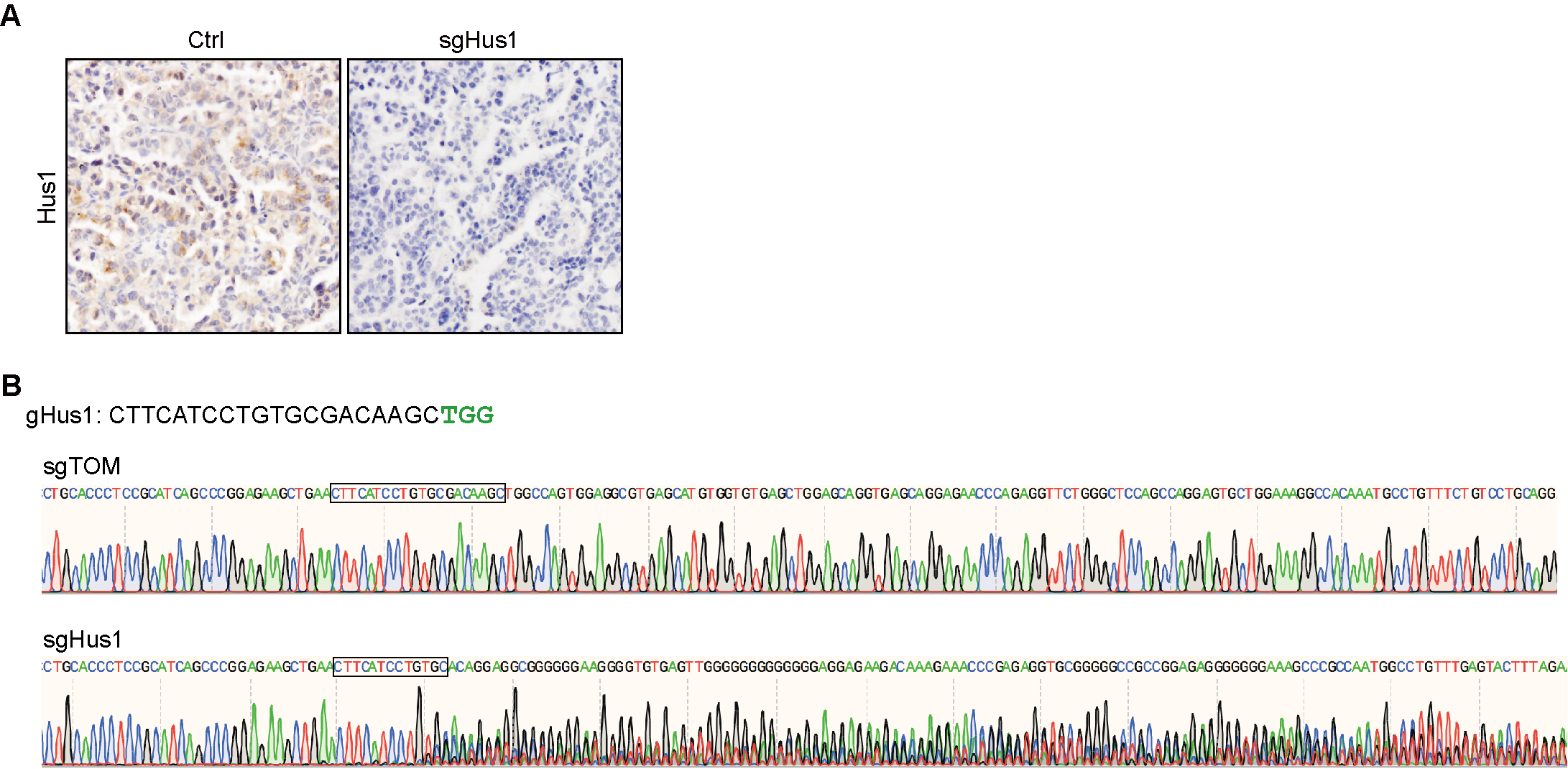

Supplement: S5 Fig — (A) Representative photographs of hematoxylin and eosin (H&E) staining of mouse lungs from control (sgTOM) and sgHus1 KP mouse. (B) The on-target efficiency of sgRNA-Hus1 in control and sgHus1 KP13 cell line. (TIF) [file pgen.1009168.s005.tif]
