# Supplementary material for: In vivo miRNA knockout screening identifies miR-190b as a novel tumor suppressor
Source: PLoS Genet. 2020 Nov 2;16(11):e1009168. doi: 10.1371/journal.pgen.1009168 (PMC7660552; doi:10.1371/journal.pgen.1009168)
Supplement: S1 Table — (PDF) [file pgen.1009168.s006.pdf]

S1 Table. A list of 16 tumor-suppresive miRNA candidates in human lung cancer.

Human miRNA ID	Murine miRNA ID	Downregulated in LC vs NL	Downregulated at T2-4 vs T1
has-let-7a-2	mmu-let-7a-2	Yes	No
has-let-7b	mmu-let-7b	Yes	No
has-let-7c	mmu-let-7c-2	Yes	No
has-miR-30b	mmu-mir-30b	No	Yes
has-miR-130a	mmu-mir-130a	No	Yes
has-miR-133b	mmu-mir-133b	Yes	No
has-miR-144	mmu-mir-144	Yes	No
has-miR-145	mmu-mir-145b	Yes	No
has-miR-146a	mmu-mir-146a	No	Yes
has-miR-150	mmu-mir-150	No	Yes
has-miR-184	mmu-mir-184	Yes	No
has-miR-190	mmu-mir-190b	Yes	No
has-miR-195	mmu-mir-195b	Yes	No
has-miR-378	mmu-mir-378d	Yes	No
has-miR-451	mmu-mir-451b	Yes	No
has-miR-497	mir-497b	No	Yes

LC:lung cancer; NL:adjacent pathologically normal lung.
