# Supplementary material for: In vivo miRNA knockout screening identifies miR-190b as a novel tumor suppressor
Source: PLoS Genet. 2020 Nov 2;16(11):e1009168. doi: 10.1371/journal.pgen.1009168 (PMC7660552; doi:10.1371/journal.pgen.1009168)
Supplement: S2 Table — (PDF) [file pgen.1009168.s007.pdf]

S2 Table. Primers used for this study.

Primer	Species	Application	Sequence
let-7a-2 sg1-F	mouse	sgRNA	CACCGAGAACGAGCTATGATTTCTC
let-7a-2 sg1-R	mouse	sgRNA	AAACGAGAAATCATAGCTCGTTCTC
let-7a-2 sg2-F	mouse	sgRNA	CACCGACAACCTACTACCTCAACCT
let-7a-2 sg2-R	mouse	sgRNA	AAACAGGTTGAGGTAGTAGGTTGTC
let-7b sg1-F	mouse	sgRNA	CACCGACCCTGCGGTGTCCGTGTTC
let-7b sg1-R	mouse	sgRNA	AAACGAACACGGACACCGCAGGGTC
let-7b sg2-F	mouse	sgRNA	CACCGGTTGTATAGTTATCTTCGG
let-7b sg2-R	mouse	sgRNA	AAACCCGAAGATAACTATACAACC
let-7c-2 sg1-F	mouse	sgRNA	CACCGTGTATAGTTACCGCAGAGCG
et-7c-2 sg1-R	mouse	sgRNA	AAACCGCTCTGCGGTAACTATACAC
let-7c-2 sg2-F	mouse	sgRNA	CACCGAGTCAGTCTTGGCGTAGAGC
let-7c-2 sg2-R	mouse	sgRNA	AAACGCTCTACGCCAAGACTGACTC
miR-30b sg1-F	mouse	sgRNA	CACCGCTGTCATACATGCGTTGGC
miR-30b sg1-R	mouse	sgRNA	AAACGCCAACGCATGTATGACAGC
miR-30b sg2-F	mouse	sgRNA	CACCGATGTTTACGTCAGCTGTCT
miR-30b sg2-R	mouse	sgRNA	AAACAGACAGCTGACGTAAACATC
miR-130a sg1-F	mouse	sgRNA	CACCGCCGAGCAGTGCAATGTTAAA
miR-130a sg1-R	mouse	sgRNA	AAACTTTAACATTGCACTGCTCGGC
miR-130a sg2-F	mouse	sgRNA	CACCGGCCGGCATGCCTTTGCTGC
miR-130a sg2-R	mouse	sgRNA	AAACGCAGCAAAGGCATGCCGGCC
miR-133a-2 sg1-F	mouse	sgRNA	CACCGATCAATGCGCAGCTACAGC
miR-133a-2 sg1-R	mouse	sgRNA	AAACGCTGTAGCTGCGCATTGATC
miR-133a-2 sg2-F	mouse	sgRNA	CACCGAACCAAATCAGCTGTTGGA
miR-133a-2 sg2-R	mouse	sgRNA	AAACTCCAACAGCTGATTTGGTTC
miR-133b sg1-F	mouse	sgRNA	CACCGACCTCTCAGGAAGACGGACT
miR-133b sg1-R	mouse	sgRNA	AAACAGTCCGTCTTCCTGAGAGGTC
miR-133b sg2-F	mouse	sgRNA	CACCGGCTGGCAAAGCTCAATATT
miR-133b sg2-R	mouse	sgRNA	AAACAATATTGAGCTTTGCCAGCC
miR-144 sg1-F	mouse	sgRNA	CACCGTATAGATGATGTACTAGTC
miR-144 sg1-R	mouse	sgRNA	AAACGACTAGTACATCATCTATAC
miR-144 sg2-F	mouse	sgRNA	CACCGATATGATGATATCCCAGCCA
miR-144 sg2-R	mouse	sgRNA	AAACTGGCTGGGATATCATCATATC
miR-145b sg1-F	mouse	sgRNA	CACCGCTGGACTGACTTCAACAGGG
miR-145b sg1-R	mouse	sgRNA	AAACCCCTGTTGAAGTCAGTCCAGC
miR-145b sg2-F	mouse	sgRNA	CACCGACCAATTTCTCAAGCCAGAC
miR-145b sg2-R	mouse	sgRNA	AAACGTCTGGCTTGAGAAATTGGTC
miR-146a sg1-F	mouse	sgRNA	CACCGCTGGGATAGCTCTGTCATCA
miR-146a sg1-R	mouse	sgRNA	AAACTGATGACAGAGCTATCCCAGC

miR-146a sg2-F	mouse	sgRNA	CACCGTCTGACATTGATATAACCCA
miR-146a sg2-R	mouse	sgRNA	AAACTGGGTTATATCAATGTCAGAC
miR-150 sg1-F	mouse	sgRNA	CACCGACAGCACTGGTACAAGGGTT
miR-150 sg1-R	mouse	sgRNA	AAACAACCCTTGTACCAGTGCTGTC
miR-150 sg2-F	mouse	sgRNA	CACCGCTATCCCCCAGGCCTGTACC
miR-150 sg2-R	mouse	sgRNA	AAACGGTACAGGCCTGGGGGATAGC
miR-184 sg1-F	mouse	sgRNA	CACCGAACTGATAAGGGTAGGTGAC
miR-184 sg1-R	mouse	sgRNA	AAACGTCACCTACCCTTATCAGTTC
miR-184 sg2-F	mouse	sgRNA	CACCGTCTGTCCGGAGAGAATCAT
miR-184 sg2-R	mouse	sgRNA	AAACATGATTCTCTCCGGACAGAC
miR-190b sg1-F	mouse	sgRNA	CACCGAGGCAAACTCTGGTCGTCAT
miR-190b sg1-R	mouse	sgRNA	AAACATGACGACCAGAGTTTGCCTC
miR-190b sg2-F	mouse	sgRNA	CACCGAGTATGCTTGACATTCAGT
miR-190b sg2-R	mouse	sgRNA	AAACACTGAATGTCAAGCATACTC
miR-195a sg1-F	mouse	sgRNA	CACCGAGAAGGGGCCAAGCCCCGA
miR-195a sg1-R	mouse	sgRNA	AAACTCGGGGCTTGGCCCCTTCTC
miR-195a sg2-F	mouse	sgRNA	CACCGGAAGTGAGTCTGCCAATAT
miR-195a sg2-R	mouse	sgRNA	AAACATATTGGCAGACTCACTTCC
miR-195b sg1-F	mouse	sgRNA	CACCGTAATTCAAGCCAAAGCTGT
miR-195b sg1-R	mouse	sgRNA	AAACACAGCTTTGGCTTGAATTAC
miR-195b sg2-F	mouse	sgRNA	CACCGAGAATTGTTACAGACCTGAA
miR-195b sg2-R	mouse	sgRNA	AAACTTCAGGTCTGTAACAATTCTC
miR-378d sg1-F	mouse	sgRNA	CACCGAAGAGATGCTGGGATAGCAC
miR-378d sg1-R	mouse	sgRNA	AAACGTGCTATCCCAGCATCTCTTC
miR-378d sg2-F	mouse	sgRNA	CACCGAGTCAGAAGGTTTGTGATT
miR-378d sg2-R	mouse	sgRNA	AAACAATCACAAACCTTCTGACTC
miR-451a sg1-F	mouse	sgRNA	CACCGTGTGGCACTTGGGAATGGCG
miR-451a sg1-R	mouse	sgRNA	AAACCGCCATTCCCAAGTGCCACAC
miR-451a sg2-F	mouse	sgRNA	CACCGACTGAGTTTAGTAATGGTAA
miR-451a sg2-R	mouse	sgRNA	AAACTTACCATTACTAAACTCAGTC
miR-451b sg1-F	mouse	sgRNA	CACCGACTGAGTTTAGTAATGGTAA
miR-451b sg1-R	mouse	sgRNA	AAACTTACCATTACTAAACTCAGTC
miR-451b sg2-F	mouse	sgRNA	CACCGCCAAGAAGAGCTCATGACCC
miR-451b sg2-R	mouse	sgRNA	AAACGGGTCATGAGCTCTTCTTGGC
miR-497b sg1-F	mouse	sgRNA	CACCGCTGTGGTTTGTACGGCACTG
miR-497b sg1-R	mouse	sgRNA	AAACCAGTGCCGTACAAACCACAGC
miR-497b sg2-F	mouse	sgRNA	CACCGTGGTGTTAGAGCGAGGGTA
miR-497b sg2-R	mouse	sgRNA	AAACTACCCTCGCTCTAACACCAC
miR-190b sg-1 F	human	sgRNA	CACCGCTGTGTGATATGTTTGATAT
miR-190b sg-1 R	human	sgRNA	AAACATATCAAACATATCACACAGC

miR-190b sg-2 F	human	sgRNA	CACCGAAACATATTCTTACAGCAGC
miR-190b sg-2 R	human	sgRNA	AAACGCTGCTGTAAGAATATGTTTC
HUS1 sg-F	mouse	sgRNA	CACCGCTTCATCCTGTGCGACAAGC
HUS1 sg-R	mouse	sgRNA	AAACGCTTGTCGCACAGGATGAAGC
qPCR-HUS1-F	human	qPCR	GAATGCCAGGGCTTTGAAAATC
qPCR-HUS1-R	human	qPCR	CACAATGCGGCTACTGCTTG
qPCR-Hus1-F	mouse	qPCR	AACACTTTCCCTGTCTTACCGT
qPCR-Hus1-R	mouse	qPCR	TCTGGGATGGAGGGTTCTTGT
qPCR-miR-190b-5p-F	mouse	qPCR	CGGCGGTGATATGTTTGATAT
qPCR-miR-146a-5p-F	mouse	qPCR	CGGCGGTGAGAACTGAATTCCA
qPCR-miR-30b-5p-F	mouse	qPCR	CGGCGGTGTAAACATCCTACAC
qPCR-miR-190b-5p-F	human	qPCR	CGGCGGTGATATGTTTGATAT
Universal-R	human/mouse	qPCR	CTGGTGTCGTGGAGTCGGCAATTC
U6-F	human/mouse	qPCR	CTCGCTTCGGCAGCACA
U6-R	human/mouse	qPCR	AACGCTTCACGAATTTGCGT
Actin-F	human/mouse	qPCR	GTCGACAACGGCTCCGGCATGTG
Actin-R	human/mouse	qPCR	CCTCTCTTGCTCTGGGCCTCGTC
miR-190b-F	human	over-expression	GCTCTAGAGCCAACGTGATCCAGCAAAGG
miR-190b-R	human	over-expression	CGGGATCCGTATAAGGGGACAAGAAATAG
miR-190b-F	mouse	over-expression	GCTCTAGAATCCAGCACACACTGAACACA
miR-190b-R	mouse	over-expression	CGGGATCCGCCTTTAGTCCCAGTGCTCAG
HUS1-F	human	over-expression	GCTCTAGAGCCACCATGAAGTTTCGGGCCAAGATC
HUS1-R	human	over-expression	ATTTGCGGCCGCGACAGGGTGCTAGGACAGCGC
RT-miR-190b-5p	mouse	Reverse transcription	CTCAACTGGTGTCGTGGAGTCGGCAATTCAGTTGAGTGATATTGGGTT
RT-miR-146a-5p	mouse	Reverse transcription	CTCAACTGGTGTCGTGGAGTCGGCAATTCAGTTGAGCATGGGTT
RT-miR-30b-5p	mouse	Reverse transcription	CTCAACTGGTGTCGTGGAGTCGGCAATTCAGTTGAGACTCAGCT
RT-miR-190b-5p	human	Reverse transcription	CTCAACTGGTGTCGTGGAGTCGGCAATTCAGTTGAGAACCCAAT
Hus1-on-target-F	mouse	on-target	GGTTGTCAGTTGTCTTCTGAG
Hus1-on-target-R	mouse	on-target	TCCTATCTCTGCCTCCTGAGT
